# Resting State Electroencephalography (EEG) Reveals Atypical Oscillatory Power in Children With Development Coordination Disorder (DCD)

**DOI:** 10.1111/psyp.70084

**Published:** 2025-06-04

**Authors:** Jarrad A. G. Lum, Kaila Hamilton, Ian Fuelscher, Pamela Barhoun, Frederik J. A. Deconinck, Arthur De Raeve, Talitha C. Ford, Tim Silk, Peter G. Enticott, Gayatri Kumar, Dwayne Meaney, Mugdha Mukherjee, Jessica Waugh, Christian Hyde

**Affiliations:** ^1^ Cognitive Neuroscience Unit, School of Psychology Deakin University Burwood Australia; ^2^ Department of Movement and Sports Sciences Ghent University Ghent Belgium; ^3^ Developmental Imaging Murdoch Children's Research Institute Parkville Australia

**Keywords:** children, developmental coordination disorder (DCD), developmental disorders, resting state electroencephalography (EEG), resting state power

## Abstract

Children with developmental coordination disorder (DCD) present with clinically significant motor impairments. Previous research indicates altered brain activity in DCD during the completion of motor and cognitive tasks, but little is known about intrinsic or spontaneous neural activity in children with the disorder. To address this gap, this study examined resting‐state electroencephalography (EEG) in 31 children with DCD and 52 age‐matched typically developing (TD) controls in both eyes‐open and eyes‐closed conditions. The mean age of the sample was 9.5 years (SD = 2.4; range 5.1–14.8). Differences in resting‐state oscillatory power between the two groups were examined in delta, theta, alpha, beta, and gamma frequency bands. Children with DCD exhibited significantly lower alpha power and higher delta power compared to the TD children in both resting state conditions. No significant differences were found in other frequency bands. Further analyses revealed that individual differences in motor functioning correlated with resting‐state alpha and delta power for the DCD, but not control group. These results suggest that intrinsic brain activity is affected in children with DCD. It is proposed that reduced alpha power and elevated delta power in DCD indicate heightened neural excitability and suboptimal neural homeostatic regulation, which may be related to the motor problems in the disorder.

## Abstract 2

We used resting‐state EEG to examine intrinsic brain activity in DCD. Children with DCD showed reduced resting‐state alpha power and elevated delta power. Resting‐state brain activity correlated with motor skills. Alpha and delta band differences might indicate suboptimal neural regulation in DCD.

## 1 Introduction

Children with developmental coordination disorder (DCD) exhibit clinically significant difficulties with fine and gross motor skills in the absence of an identifiable medical or neurological condition (American Psychiatric Association 2013). Recent estimates suggest that the disorder affects approximately 5% of children (Li et al. 2024). These motor difficulties negatively impact children's quality of life by interfering with everyday tasks such as dressing, writing or typing, using cutlery, and participating in sports (Magalhães et al. 2011; Summers et al. 2008; Van der Linde et al. 2015). Beyond the motor domain, growing evidence suggests that these difficulties may also be associated with increased mental health concerns (Tamplain and Miller 2021). Although DCD is primarily characterized as a movement disorder, impairments in attention, language, memory, reading, and social functioning are also commonly reported in this population (Alloway 2007; Archibald and Alloway 2008; Dewey et al. 2002; Farran et al. 2020; Iversen et al. 2005). DCD frequently co‐occurs with other neurodevelopmental disorders, most notably attention‐deficit/hyperactivity disorder (ADHD), with comorbidity rates estimated at around 50% (Goulardins et al. 2017). This heterogeneity complicates both identification and treatment planning.

A growing body of evidence suggests that DCD is associated with altered brain functioning (e.g., Subara‐Zukic et al. 2022; Zwicker 2021). Functional magnetic resonance imaging studies have shown atypical activation in the frontal lobes, parietal lobes, cerebellum, and basal ganglia during cognitive and sensorimotor processing (Biotteau et al. 2016; Fuelscher et al. 2018; Wilson et al. 2017; Zwicker et al. 2012). However, little is known about intrinsic or spontaneous neural activity in this disorder. This type of brain activity, observed in task‐free or resting‐state paradigms (Llinás 2002), reflects the brain's functional architecture, which in turn influences the processing of sensory‐motor information, completion of higher‐order operations (Deco and Corbetta 2011; Raichle 2015; Sadaghiani and Kleinschmidt 2013), and acquisition of new motor skills (Lum et al. 2023; Mary et al. 2017; Wu et al. 2014). While altered spontaneous neural activity appears to be present in a range of neurodevelopmental disorders (e.g., Newson and Thiagarajan 2019), it remains unclear whether this is also the case in DCD. The current study addressed this gap in the literature by examining resting‐state electroencephalography (EEG) in children with DCD.

In resting‐state research, brain activity is recorded as participants either look at a benign stimulus on a computer display (e.g., a dot) or close their eyes for a few minutes (Anderson and Perone 2018). During this time, oscillatory brain activity can be observed in EEG and magnetoencephalography signals (e.g., Barry et al. 2007). Oscillatory activity is generated from cycles of synchronized and desynchronized firing by populations of neurons. The dendritic electrical activity arising from this process results in rhythmic fluctuations, or oscillations, in the electroencephalogram and magnetoencephalogram (Cohen 2017). In humans, these oscillations occur at varying speeds, termed delta (1–3 Hz; i.e., 1–3 oscillations per second), theta (4–7 Hz; i.e., 4–7 oscillations per second), alpha (8–12 Hz; i.e., 8–12 oscillations per second), beta (13–30 Hz; i.e., 13–30 oscillations per second), and gamma (> 30 Hz; i.e., greater than 30 oscillations per second) (Buzsáki et al. 2013). Synchronization in beta, gamma, and alpha bands supports relatively fast processing of local information, while synchronization in theta and delta bands, though slower, integrates processing across different brain regions, incorporating outputs from beta and gamma networks (Buzsaki 2006; Buzsaki and Draguhn 2004; da Silva 1991; Lisman and Jensen 2013). Amplitude or “power” (i.e., amplitude‐squared) is one of the most widely used metrics to quantify intrinsic brain activity in the aforementioned frequency bands (Anderson and Perone 2018; Gasser et al. 1988; Newson and Thiagarajan 2019). Generally, as more neurons synchronously fire in a specific frequency band, power increases (Pfurtscheller and Aranibar 1977).

Atypical resting‐state power has been observed in a range of neurodevelopmental disorders (Clarke et al. 2020; Jäncke et al. 2019; Neo et al. 2023; Papagiannopoulou and Lagopoulos 2016; Stanojević et al. 2023). In this research, power in one or more frequency bands is often found to be higher or lower in the affected group compared to controls. For example, the evidence suggests that in ADHD, which commonly occurs with DCD (Goulardins et al. 2017), resting‐state power tends to be higher in the delta and theta bands and lower in the beta band compared to controls (Clarke et al. 2020; Newson and Thiagarajan 2019). In autism, another disorder associated with motor impairments (Fournier et al. 2010; Licari et al. 2020), resting‐state power tends to be lower in the alpha band but higher in the gamma band (for a recent meta‐analysis, see Neo et al. 2023). This suggests that resting oscillatory activity may subserve common behavioral traits observed in neurodevelopmental disorders.

Only a small number of studies have examined resting state power in DCD. A MEG study by Van Dyck et al. (2022) examined resting state power in just under 30 children with and without DCD. One set of analyses revealed no significant differences in resting state theta, alpha, or beta power between the groups. Another set of analyses, however, indicated DCD was associated with elevated resting state functional connectivity, which measures synchronization between cortical regions, in the alpha and beta bands. This result was primarily observed in parietal, occipital, temporal, and cerebellar regions. Correlation analyses revealed that resting state brain activity in the theta, alpha, and beta bands was negatively correlated with a measure of motor functioning. Meachon et al. (2024) recently examined resting‐state EEG in adults with DCD. The results of this study implicate alpha and beta power in the disorder. Specifically, alpha power, averaged over frontal, central, and occipital electrodes, was found to be higher in the DCD group compared to controls. Additionally, beta power was higher in DCD at frontal electrodes and lower at occipital electrodes. No significant differences were observed between the DCD and control groups in the delta, theta, and gamma bands. These results suggest that DCD may be associated with deficits in regulating cortical excitability, a process supported by alpha oscillations (Klimesch et al. 2007; Sauseng et al. 2009), as well as neural deficits in the local processing of information, including sensory processing, attention, and motor control, which are mediated by beta oscillations (Pfurtscheller and Da Silva 1999). In that study, no significant correlations were found between resting‐state power and individual differences in motor functioning, making it unclear whether atypical resting‐state power in DCD is specifically related to the level of motor functioning in adults.

### 1.1 The Current Study

The current study further investigated intrinsic neural activity in children with DCD. We acquired resting‐state EEG data from 31 children with DCD and 52 age‐matched typically developing (TD) controls under both eyes‐open and eyes‐closed conditions. Acquiring resting‐state data while participants have their eyes open likely reflects intrinsic activity related to visual sensory and online cognitive processing. In contrast, acquiring the data with eyes closed may reflect processes related to supporting the internal regulation of brain activity since the data is obtained under conditions of reduced sensory input (Anderson and Perone 2018; Barry et al. 2009, 2007; Petro et al. 2022). Our analyses first tested for differences in resting‐state power between DCD and TD groups in the delta, theta, alpha, beta, and gamma frequency bands. Based on past resting‐state DCD research (Meachon et al. 2024; Van Dyck et al. 2022), we hypothesized that alpha power would be higher in the DCD group. Differences between groups were also hypothesized in the beta band, with DCD associated with higher power in this frequency band. Additionally, we conducted exploratory correlation analyses to determine whether resting‐state power was associated with motor functioning in the DCD and TD groups.

## 2 Method

### 2.1 Participants

A total of 31 children with DCD and 52 typically developing (TD) children participated in the study (see Table 1 for a summary of demographic information). The sample size for each group was determined by the number of children who met the criteria for inclusion in either the DCD or control group (as described below) and from whom we were able to obtain relatively noise‐free resting‐state EEG data. Participants were recruited via flyers and through occupational therapists. Written consent was obtained from the parents of each child, and assent was obtained from the children. Families received financial reimbursement for their time. The Deakin University Human Research Ethics Committee approved the project.

**TABLE 1:** Demographic and clinical characteristics of children with developmental coordination disorder (DCD) and typically developing (TD) controls.

Variable	DCD		TD		*p*
	*M* or %	SD	*M* or %	SD	
% Female	59.4%	—	46.2%	—	0.242^c^
% Right‐handed	84.4%	—	92.3%	—	0.232^c^
Age (years; months)	9; 5	2; 6	9; 6	2; 5	0.932^d^
BOT‐2 SF^a^	35.9 (9th percentile)	3.5	53.0 (59th percentile)	6.6	< 0.001^d^
DCD‐Q (total score)^a^	35.7	9.4	59.8	12.6	< 0.001^d^
ADHD RS‐IV (total score)^b^	20.0	11.8	16.2	14.0	0.125^d^
WASI‐2 Matrix Reasoning^a^	46.7	11.4	56.0	10.3	< 0.001^d^
WASI‐2 Vocabulary^a^	49.2	11.6	53.8	9.3	0.055^d^

Abbreviations: ADHD RS‐V = Attention Deficit Hyperactivity Disorder Rating Scale; BOT‐2 = Bruininks‐Oseretsky Test of Motor Proficiency 2nd Edition; DCDQ = Developmental Coordination Disorder Questionnaire; WASI‐2 = Wechsler Abbreviated Scale of Intelligence‐2nd Edition.

^a^ Data shown in the table are *T*‐scores (i.e., mean of 50 & standard deviation of 10).

^b^ Data shown in the table are raw scores (range 0–54). Higher scores indicate greater severity of ADHD symptoms.

^c^ Fischer's exact test evaluated differences in frequencies.

^d^ Mann–Whitey *U* tests evaluated differences in group means.

Children with DCD were screened against the DSM‐5 definition and met Criteria A–D (American Psychiatric Association 2013). All children with DCD presented with below age‐appropriate motor skills (Criterion A), as indicated by a score at or below the 16th percentile on the short form of the Bruininks‐Oseretsky Test of Motor Proficiency 2nd edition (BOT‐2 SF; Bruininks and Bruininks 2005), a standardized test of fine and gross motor skills. The internal consistency (Cronbach's Alpha) of the BOT‐2 SF overall score for ages 4–7 years, 8–11 years, and 12–21 years is 0.82, 0.83, and 0.86, respectively (Bruininks and Bruininks 2005). Next, the motor difficulties identified above interfered with the child's ability to perform daily activities involving movement (Criterion B), as indicated by scores on the Developmental Coordination Disorder Questionnaire (DCD‐Q; Wilson et al. 2009). The DCD‐Q is a parental report survey that assesses the extent to which motor impairments impact daily functioning. The DCD‐Q has demonstrated high internal consistency, with Cronbach's *α* = 0.89 (Wilson et al. 2009). In the absence of Australian norms, we used an approach adopted in our earlier work to define functional motor impairment (see Barhoun et al. 2021; Bianco et al. 2024; Hyde et al. 2014, 2018). That is, based on DCD‐Q scores of all control children from the larger study (Bianco et al. 2024) from which our sample was derived, we identified the 95% confidence intervals (CI_95%_) for the DCD‐Q scores for those aged 5–7 (CI_95%_: 59.75 ± 3.48) aged 8–9 (CI_95%_: 64.00 ± 4.44), and aged 10–14 (CI_95%_: 65.05 ± 4.02). All children in the DCD group scored below the lower arm of the 95% confidence interval for their age band, thereby meeting Criterion B (i.e., they presented with motor‐related deficits of everyday living). Further, since participants in the study were children, we established that the onset of motor impairments occurred during childhood (Criterion C). Finally, motor skill difficulties were not otherwise attributable to any medical or neurodevelopmental disorder (Criterion D). Children in the control group had age‐appropriate motor ability, all scoring above the 16th percentile on the BOT‐2 SF (Bruininks and Bruininks 2005) and DCD‐Q, and had not been clinically diagnosed with a neurodevelopmental disorder.

Additionally, given the common co‐occurrence between DCD and ADHD (Kadesjö and Gillberg 1998), we measured the severity and frequency of attentional difficulties in the sample using the ADHD Rating Scale‐IV (ADHD‐RS‐IV; DuPaul et al. 1998). The ADHD‐RS‐IV measures overall ADHD symptoms. In this study, we used raw scores, which range from 0 to 54, with higher scores indicating greater severity/frequency of ADHD symptoms. Internal consistency for the AHD‐RS‐IV total score has been found to be 0.94 (Pappas 2006). Data from this instrument were used as a covariate in the correlation analyses. Finally, we assessed children's general cognitive functioning using the “Matrix Reasoning” and “Vocabulary” subtests from the Wechsler Abbreviated Scale of Intelligence‐2nd Edition (WASI‐II; Wechsler 2011). The Matrix Reasoning subtest assesses visuo‐spatial reasoning skills, and the Vocabulary subtest assesses semantic knowledge. The average split‐half reliability for the Matrix Reasoning and Vocabulary subtests across 6–16‐year‐olds is 0.87 and 0.91, respectively (Wechsler 2011). Performance on both WASI‐II subtests is described by a *T*‐score.

Table 1 presents summary statistics from standardized tests along with the age, gender, and handedness distributions for each group. This table also includes results from statistical tests comparing differences between groups on all variables. As expected, significant differences between the groups were observed on the DCD‐Q and BOT‐2 SF, with those with DCD performing significantly lower in each case (*p* < 0.05). The differences between groups with respect to age, gender, handedness, ADHD RS‐IV, and the Vocabulary subtest from the WASI‐II were all non‐significant. The DCD group obtained significantly lower scores on the Vocabulary and Matrix Reasoning subtests from the WASI‐II. The difference between groups on these subtests was larger for Matrix Reasoning. This result likely reflects visuo‐spatial difficulties associated with the disorder (Wilson and McKenzie 1998).

### 2.2 Resting State EEG

Resting‐state EEG data were acquired in both “eyes‐open” and “eyes‐closed” conditions. Before data acquisition, an elastic EEG cap (EasyCap, Germany) was fitted to each child's head. Embedded in the cap were 23 Ag/AgCl electrodes positioned according to the 10/20 system. Specifically, electrodes were placed at Fp1, Fp2, F9, F7, F3, AFz, F10, Fz, F4, F8, M1 (left mastoid), T7, C3, Cz, C4, T8, M2 (right mastoid), P7, P3, Pz, P4, P8, O1, and O2 locations. After reducing impedances to under 10 kΩ, resting‐state data in the eyes‐open condition were acquired. For this condition, children were asked to focus on a fixation point, a “+” sign, displayed on a 17‐in. computer monitor for 90 s. Next, resting‐state data in the eyes‐closed condition were collected, during which the children closed their eyes for 90 s. A 90‐s resting state protocol was selected since it placed minimal demands on children's attentional skills, thereby reducing movement artifacts in the EEG data. Additionally, evidence from one meta‐analysis found no difference in resting state power between studies that used 90‐s and 10‐min resting state protocols in children with ADHD (Arns et al. 2013). Finally, both conditions were completed with minimal noise; computer fans were the only auditory stimulation present.

### 2.3 Procedure

All children were individually tested in a laboratory setting in a single session lasting approximately 3 hours. During the first part of the session, children were administered a battery of standardized tests, including the BOT‐2 and WASI‐2. After the completion of these tasks, resting state EEG data were then acquired from the children. Concurrently, during the test session, parents completed a demographic survey, DCD‐Q, and ADHD RS‐IV.

### 2.4 Electroencephalography

#### 2.4.1 EEG Data Acquisition

EEG signals were recorded using a tMSI RefA amplifier (Twente Medical Systems International, The Netherlands) in conjunction with Polybench software (Version 1.30; Twente Medical Systems International, The Netherlands). Data were sampled at 2048 Hz with a common average reference, and electrode AFz was used as the ground. No online filters were applied during acquisition. As noted above, electrode impedances were kept below 10 kΩ prior to recording.

#### 2.4.2 EEG Pre‐Processing

Pre‐processing of EEG data from the eyes‐open and eyes‐closed conditions was conducted using EEGLAB (Version 10.04; Delorme and Makeig 2004) run in MATLAB (Version 2023a). Data were first down‐sampled to 1000 Hz. Next, a series of zero‐phase FIR filters were applied: a high‐pass filter with a 0.5 Hz cutoff and a transition band of approximately 0.125 Hz (Hamming window), a notch filter centered at 50 Hz to remove line noise using a Parks‐McClellan optimal equiripple design with a filter order of 180, and a low‐pass filter with a cutoff of 80 Hz and a transition band of approximately 20 Hz (Hamming window). All filters were applied non‐causally to prevent phase distortion. The EEG data were then visually inspected for movement artifacts, leading to short bursts of noise present across all channels. These types of artifacts, lasting no more than 1–2 s, were removed via deletion. The EEG data were then adjusted for oculomotor artifacts using ICLabel (Pion‐Tonachini et al. 2019). Specifically, the EEG data were first submitted to independent components analysis (using the Infomax algorithm), and ICLabel was used to identify “eye” components, which capture blinks and eye movements. This component was only removed if the ICLabel algorithm identified it as an eye artifact with at least 90% probability. After data pre‐processing, electrodes FP1, FP2, F9, and F10 were excluded from further analysis since their inclusion was only to capture eye movements and blinks. Data from all electrodes were then re‐referenced to the average of the left and right mastoids. The electrodes submitted for spectral analysis were F7, F3, Fz, F4, F8, T7, C3, Cz, C4, T8, P7, P3, Pz, P4, P8, O1, and O2.

There were no significant differences between groups in the duration of the resting‐state recording after artifact rejection. Following pre‐processing, the mean duration of retained EEG data (out of a maximum of 90 s) for the DCD group was 85.5 s (SD = 8.6) in the eyes‐open condition and 86.8 s (SD = 9.5) in the eyes‐closed condition. For the TD group, the mean duration was 86.7 s (SD = 7.6) in the eyes‐open condition and 88.9 s (SD = 8.1) in the eyes‐closed condition. Mann–Whitney *U* tests revealed no significant group differences in the amount of usable data after artifact rejection for either the eyes‐open (*z* = 0.580, *p* = 0.562) or eyes‐closed condition (*z* = 0.927, *p* = 0.354).

#### 2.4.3 Spectral Analysis

**FIGURE 1:**
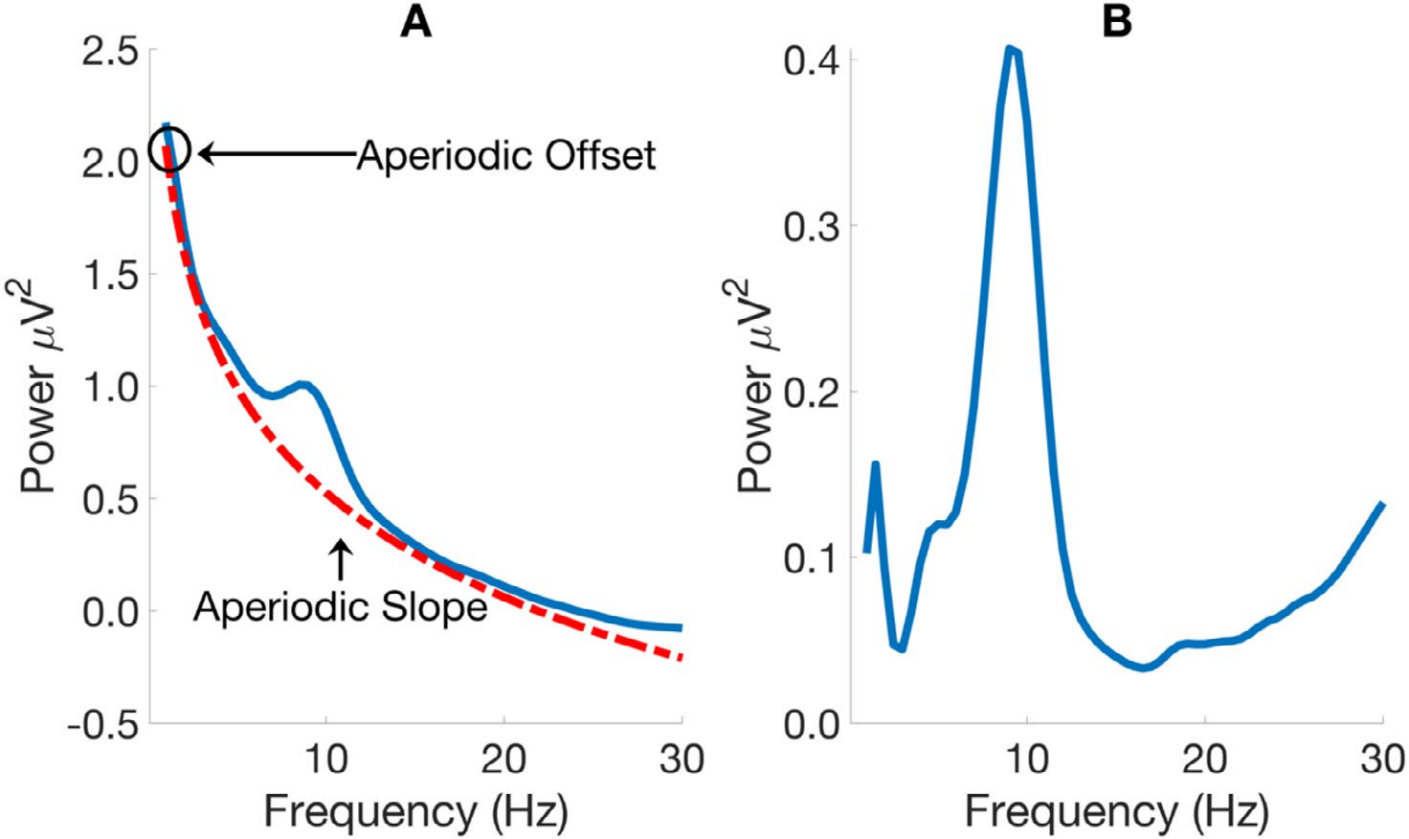
Separating periodic and aperiodic parameters in the data. Panel (A) shows the power spectra from Welch's Method averaged across all participants, electrodes, and resting state conditions before removing aperiodic activity. In this panel, the dashed line indicates the aperiodic components of the power spectrum computed by the FOOOF algorithm. Panel (B) shows the power spectrum after removing aperiodic parameters from the data.

Time series data at each electrode were converted to the frequency domain using Welch's Method (2‐s window with a Hann Taper and 50% overlap window) from 0.5 to 80 Hz. The sampling rate of 1000 Hz afforded a frequency resolution of 0.5 Hz without zero‐padding. The amplitude at each frequency point was computed in terms of μV^2^ or power. In the next step, aperiodic activity was removed from the power spectrum using the FOOOF toolbox run in Python 3.10 via a MATLAB wrapper (Donoghue et al. 2020). The aperiodic component of the power spectrum is characterized by two parameters termed the aperiodic offset and slope. The aperiodic slope quantifies the rate at which power decreases as frequencies increase, and the offset describes power at the lowest frequency point in the power spectrum (see Panel A of Figure 1). The aperiodic slope appears to capture the ratio of excitatory to inhibitory activity in the brain and the offset, random spiking (Gao et al. 2017; Manning et al. 2009; Miller et al. 2012). To obtain accurate aperiodic parameters, a two‐step procedure was used to circumvent the effects of the 50 Hz notch filter. In the first step, the FOOOF algorithm was applied to the power spectrum from 1 to 45 Hz (FOOOF parameters: frequency range = [1, 45], peak width limits = [1, 12], maximum number of peaks = no limit, peak threshold = 2, minimum peak height = 0.0), and in the second step, the algorithm was applied to the power spectrum from 55 to 80 Hz (FOOOF parameters: frequency range = [55, 80], peak width limits = [1, 12], maximum number of peaks = no limit, peak threshold = 2, minimum peak height = 0.0).

There is a growing literature investigating the importance of resting state aperiodic parameters in terms of understanding brain functioning in clinical and non‐clinical populations (e.g., Hill et al. 2022; Ostlund et al. 2021; Shuffrey et al. 2022). We also undertook exploratory analyses examining aperiodic parameters in children with DCD. Results from these analyses revealed no significant differences between DCD and TD groups with respect to the aperiodic offset and slope. The results of these analyses are presented in the Supporting Information S1 (see Tables S1 and S2). Relevant to the current study, however, is evidence demonstrating that failure to remove aperiodic activity from the data can exaggerate associations between resting state power and behavioral/cognitive outcomes (e.g., Ouyang et al. 2020). Aperiodic activity from our data was removed by first computing the aperiodic slope and offset at each electrode and participant. Next, these aperiodic components were subtracted from the power spectrum derived from Welch's Method. This effectively removes the aperiodic parameters from the data, accentuating oscillatory activity. The effect of removing the aperiodic parameters from the signal is summarized in Figure 1. Panel A of Figure 1 shows power averaged over all participants, electrodes, and resting state conditions before removing aperiodic activity. Also shown in this panel are the aperiodic parameters computed for our data. Panel B shows the same data after aperiodic activity is removed.

### 2.5 Data Analysis

Following Meachon et al. (2024), we tested for differences between the TD and DCD groups in both the eyes‐open and eyes‐closed conditions using absolute power (after removing aperiodic parameters). Specifically, the power spectrum at each electrode was averaged into bins representing canonical frequency bands: delta (1–3 Hz), theta (4–7 Hz), alpha (8–12 Hz), beta (13–30 Hz), gamma 1 (31–40 Hz) and gamma 2 (50–80 Hz). Splitting the gamma band into lower (gamma 1) and higher (gamma 2) sub‐bands allowed us to examine potential frequency‐specific group differences while avoiding the distortion introduced by the notch filter. Differences between the groups at each of the 17 electrodes and frequency bands were evaluated using Mann–Whitney *U* tests, given that power data were non‐normally distributed and/or variances were unequal between groups. Thus, at each frequency band, a total of 17 statistical tests were undertaken (one per electrode). To control for multiple comparisons, we applied Benjamini and Hochberg's (1995) False discovery rate (FDR) procedure to each set of 17 uncorrected *p*‐values.

## 3 Results

**FIGURE 2:**
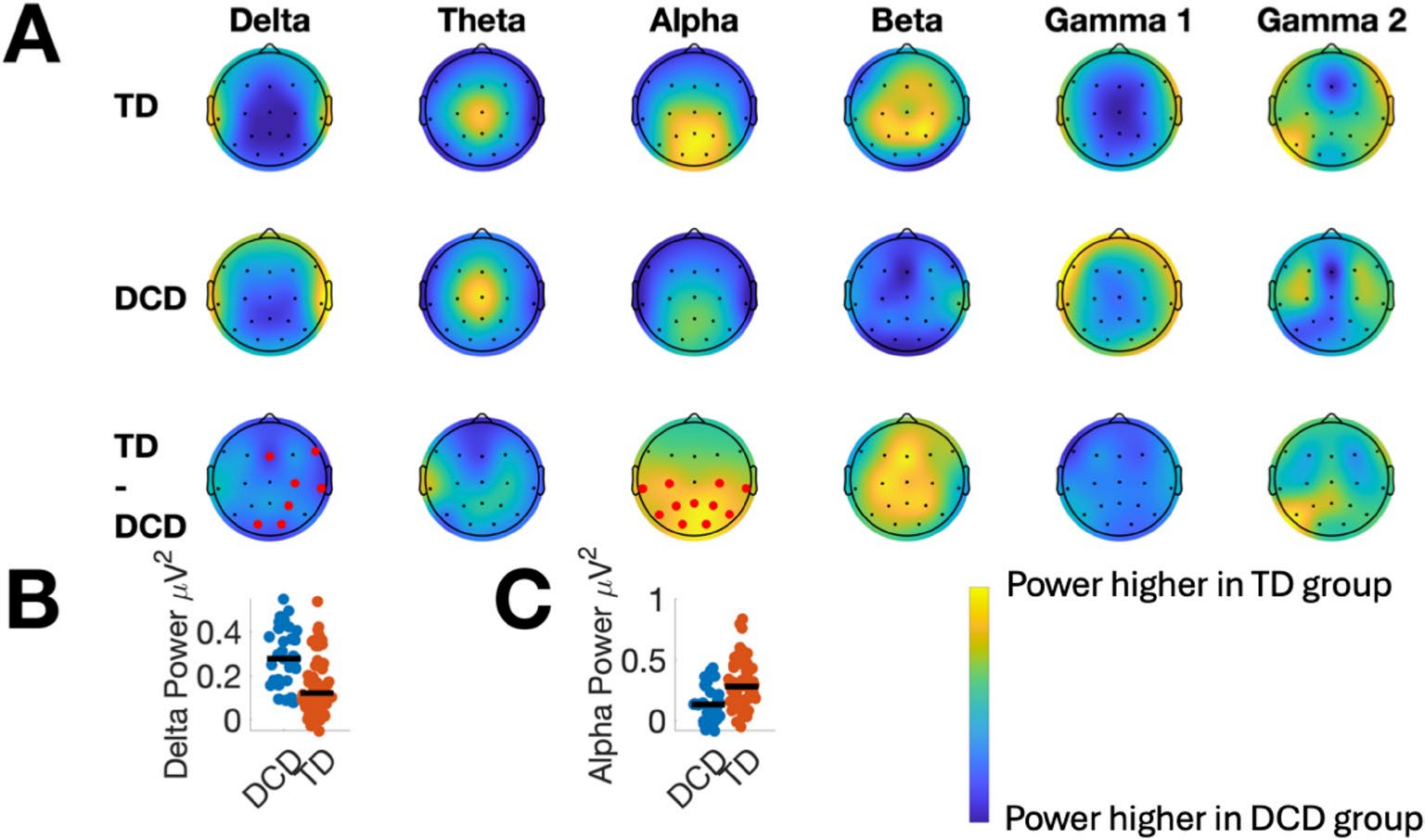
Resting state power in the delta, theta, alpha, beta, and gamma bands from the *eyes‐open* condition. The first two rows in Panel (A) show topographical plots for the TD and DCD groups, respectively. The difference in power between the groups is shown in the third row (TD—DCD). In this row, lighter colors (yellow) indicate power is higher in the TD group compared to the DCD group, and darker colors (blue) indicate power is higher in DCD. Electrodes marked in red indicate significant group differences after applying the FDR correction. Panels (B) and (C) show swarm plots illustrating individual differences in delta and alpha power for each group, respectively. In Panels B and C, power was averaged over electrodes associated with significant differences between TD and DCD groups. The black horizontal lines in Panels (B) and (C) show median power for each group. Summary statistics for all plots (including range) are presented in Table S3.

**FIGURE 3:**
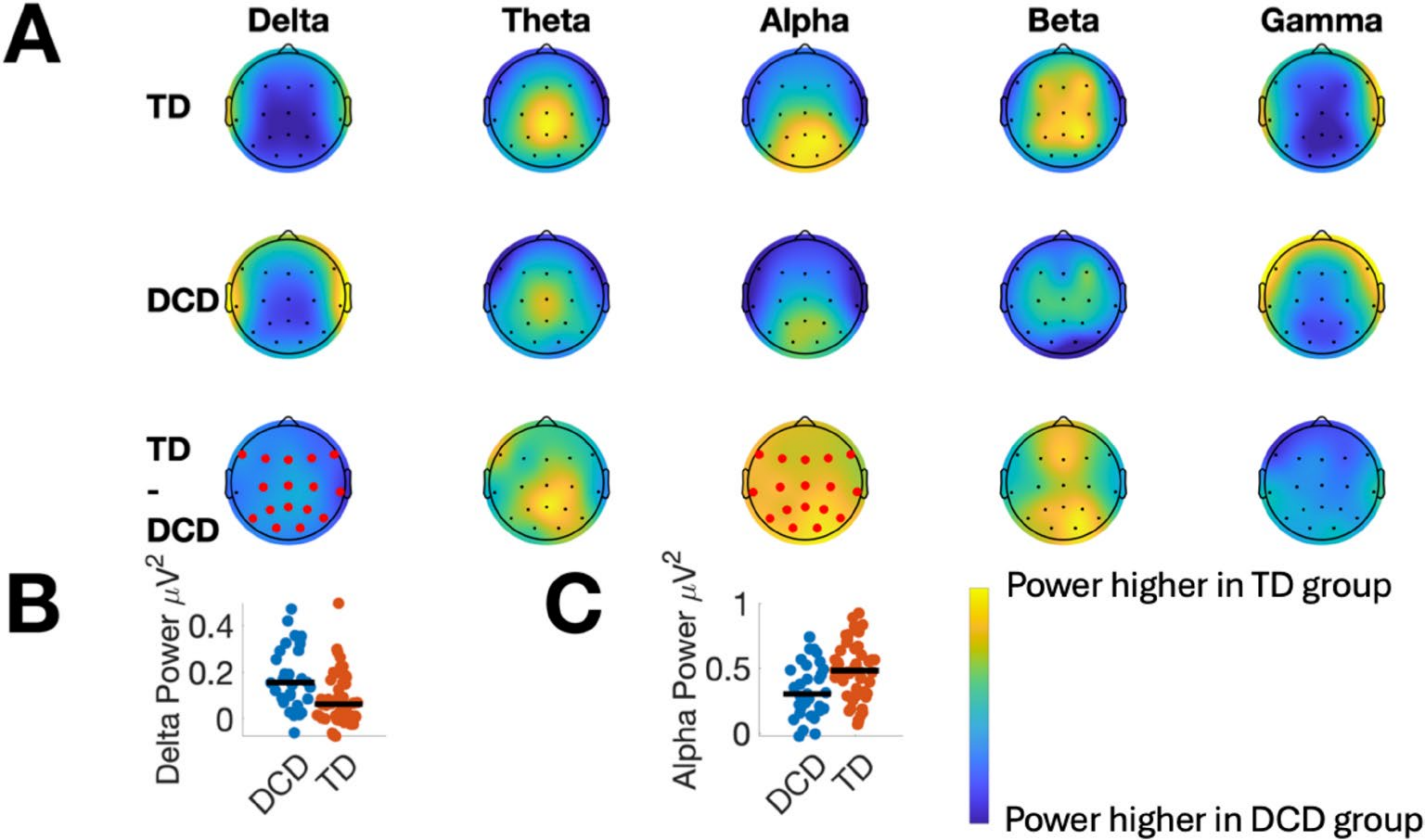
Resting state power in the delta, theta, alpha, beta, and gamma bands from the *eyes‐closed* condition. The first two rows in Panel (A) show topographical plots for the TD and DCD groups, respectively. The difference in power between the groups is shown in the third row (TD—DCD). In this row, lighter colors (yellow) indicate power is higher in the TD group compared to the DCD group, and darker colors (blue) indicate power is higher in DCD. Electrodes marked in red indicate significant group differences after applying the FDR correction. Panels (B) and (C) show swarm plots illustrating individual differences in delta and alpha power for each group, respectively. In Panels B and C, power was averaged over electrodes associated with significant differences between TD and DCD groups. The black horizontal lines in Panels (B) and (C) show median power for each group. Summary statistics for all plots (including range) are presented in Table S5.

The first set of analyses tested for differences in resting state power between the DCD and TD groups. Figure 2 presents topographical plots summarizing the distribution of delta, theta, alpha, beta, and gamma power across the scalp from the eyes‐open resting state condition. Panel A in Figure 2 shows data from the TD group (top row), DCD group (second row), and differences in power between the two groups (third row). Electrodes highlighted in red indicate a significant group difference in power after applying the FDR correction. Figure 3 presents corresponding data from the eyes‐closed condition.

In the eyes‐open condition, DCD was associated with significantly lower power in the delta and alpha bands (see Figure 2). Significant differences in delta band power were only observed at electrodes O1, O2, Fz, C4, and F8. In this frequency band, delta power was higher in the DCD group. Differences in the alpha band were observed at occipital, parietal, and central electrodes. In the alpha band, DCD was associated with lower resting‐state power. For illustrative purposes, Panels B and C in Figure 2 present swarm plots of delta and alpha power, respectively, highlighting individual differences in power for both groups. In this panel, power data were averaged over significant electrodes. No significant differences in resting‐state power between the TD and DCD groups were found in the theta, beta, or gamma bands. Table S4 presents FDR‐corrected *p*‐values comparing the DCD and TD groups at all electrodes and frequency bands in the eyes‐open condition. In the eyes‐closed condition, significant differences between the groups were observed in the delta and alpha bands at almost all electrodes. In the delta band, resting‐state power was lower in the DCD group and higher in the alpha band. Panels B and C in Figure 3 show individual differences in delta and alpha power, respectively. In the eyes‐closed condition, differences between the groups in the theta, beta, and gamma bands were all non‐significant. FDR‐corrected *p*‐values comparing the DCD and TD groups at all electrodes and frequency bands in the eyes‐closed condition are presented in Table S6.

The second set of analyses tested whether individual differences in motor functioning were associated with resting‐state EEG power within each group. Spearman's *ρ* was used to assess correlations between the average of BOT‐2 and DCD‐Q T‐scores and delta and alpha power. To improve signal‐to‐noise ratio and reduce the number of statistical comparisons, EEG power was averaged across electrodes that showed significant group differences (i.e., Panels B and C of Figures 2 and 3), and across eyes‐open and eyes‐closed conditions. Correlations were computed separately for the DCD and TD groups. To test whether the strength of associations differed between groups, we conducted Fisher's *r*‐to‐*z* transformations on each pairwise correlation. A significant group difference in correlation suggests that the neural processes reflected in that frequency band may contribute differently to motor functioning in DCD and TD children. For instance, a stronger association in the DCD group may reflect greater functional reliance on activity in that band, while similar correlations across groups would suggest a shared neurophysiological basis for motor functioning.

**FIGURE 4:**
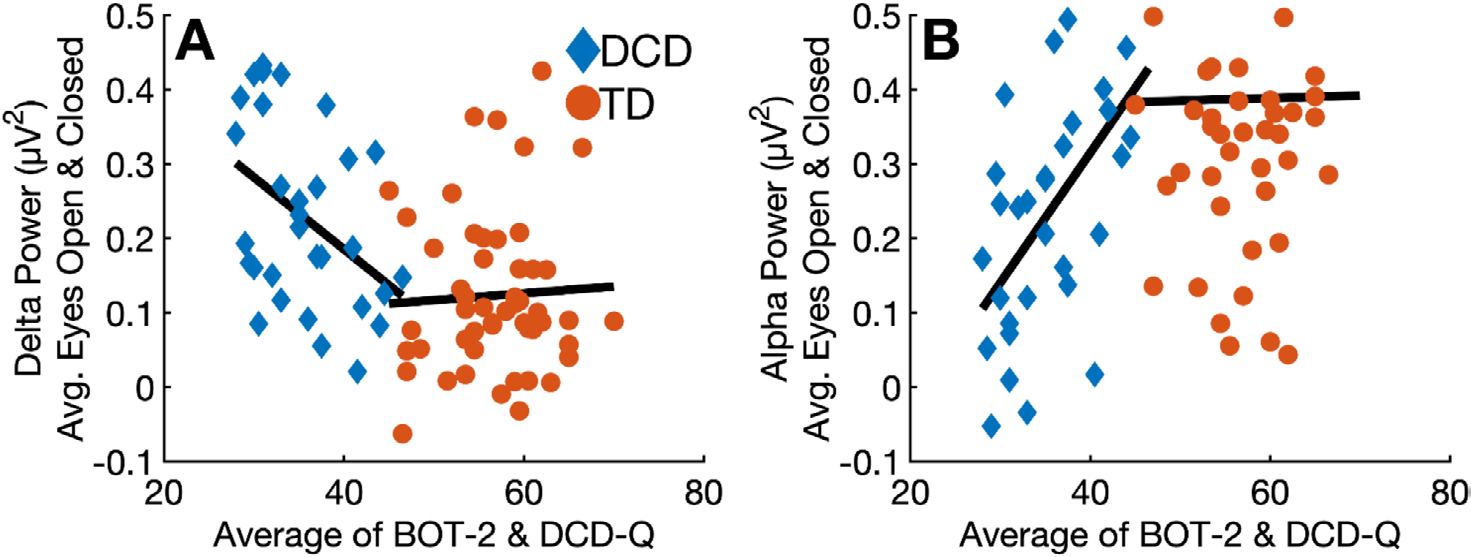
Scatterplots showing the relationship between motor functioning and resting‐state power by group. Panel (A) shows the association with delta power, and Panel (B) shows the association with alpha power.

Figure 4 presents these associations separately by group. Panels A and B show correlations between motor skills and resting‐state delta and alpha power, respectively. Resting‐state delta power was significantly negatively correlated with motor skills in the DCD (*ρ* = −0.416, *p* = 0.020), but not in the TD group (*ρ* = −0.023, *p* = 0.869). This difference in correlation strength was statistically significant (*z* = 1.968, *p* = 0.049). A similar pattern emerged for alpha power: in the DCD group, resting‐state alpha power was positively associated with motor functioning (*ρ* = 0.565, *p* < 0.001), whereas the correlation was non‐significant in the TD group (*ρ* = 0.051, *p* = 0.720). This difference between correlation strengths was also significant (*z* = 2.204, *p* = 0.028). Collectively, these results indicate that in children with DCD, poorer motor skills are associated with higher delta power and lower alpha power at rest; an association not observed in the TD group.

## 4 Discussion

This study revealed atypical resting‐state power in children with DCD. Contrary to hypotheses derived from adult research (Meachon et al. 2024), our findings showed that children with DCD exhibited lower alpha power and higher delta power at rest compared to typically developing controls. No significant differences in beta power were found between the groups. Additionally, unlike past research undertaken with adults, we found that resting‐state brain activity correlated with motor proficiency; poorer motor skills were associated with lower alpha power and higher delta power. This association appears to be specific to DCD. The correlation between delta/alpha resting‐state power and motor skills was not significant in the TD group. Collectively, these results suggest that intrinsic brain activity in children with DCD not only differs from that of age‐matched controls but may also be related to the severity of motor impairment in affected children.

Lower resting‐state alpha power in children with DCD was observed in both eyes‐open and eyes‐closed conditions. This finding suggests that atypical alpha activity in DCD could be a fundamental brain state, unaffected by the presence or absence of sensory stimulation. Furthermore, differences in alpha power between DCD and TD groups were found across nearly all electrode sites, indicating that reduced alpha power is not confined to a specific lobe or hemisphere. Alpha oscillations regulate cortical excitability; localized increases in alpha power have an inhibitory effect on cortical functioning, while decreases release regions necessary to complete a task from inhibition (Bonnefond and Jensen 2012; Händel et al. 2011; Klimesch et al. 2007; Pfurtscheller 1992; Pfurtscheller and Aranibar 1977). Our data suggest that during childhood, DCD is characterized by a heightened state of neural excitability.

There are empirical grounds to suspect that the absence of a robust alpha rhythm could disrupt motor and cognitive functions. During movement and the execution of higher‐order operations, alpha activity modulates neuronal excitability, facilitating the selective processing of relevant information while suppressing irrelevant or distracting inputs (Klimesch et al. 2007). This gating mechanism may be disrupted in DCD, leading to “neural noise” or interference from non‐target cortical regions during motor planning and execution. The significant correlation between resting state alpha power and motor functioning suggests that higher levels of resting state cortical excitability (i.e., lower alpha power) may be specifically impacting motor skills in DCD. Furthermore, since alpha oscillations are implicated in a range of higher‐order processes such as working memory (Bonnefond and Jensen 2012) and language (Lum et al. 2024), elevated resting state power in this frequency band may contribute to the presence of co‐occurring impairments in the disorder. This possibility needs to be investigated in future research by examining changes in alpha power as children with DCD complete motor and cognitive tasks. In healthy controls, alpha power decreases over cortical regions involved in a task and increases in non‐relevant regions (Pfurtscheller 1992; Pfurtscheller and Aranibar 1977; Pfurtscheller and Da Silva 1999). We suspect this modulation of alpha activity might be attenuated in DCD.

**FIGURE 5:**
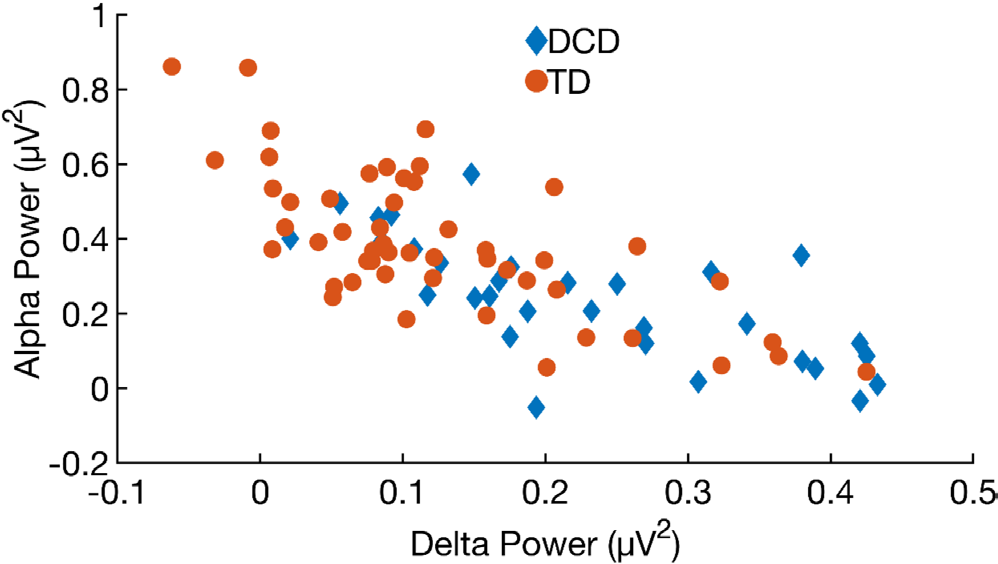
A scatterplot showing the relationship between resting state delta and alpha power averaged across eyes‐open and eyes‐closed conditions. Children with higher resting state delta power typically have lower resting state alpha power (indicating higher levels of cortical excitability).

The study also revealed elevated resting‐state delta power in children with DCD. Differences in delta power between the DCD and TD groups were more pronounced in the eyes‐closed condition. This might suggest disruptions in delta band activity are more strongly associated with offline processing of sensory/cognitive information and the regulation of internal neurological states. In particular, we suspect this finding reflects suboptimal neural homeostatic regulation in DCD, since this is one of the functions of delta oscillations (Knyazev 2012). During wakeful periods, learning and other interactions with the environment promote synaptic potentiation (Vyazovskiy et al. 2008). Synaptic downscaling is a crucial complementary process that occurs during sleep, where synaptic strength is uniformly reduced via delta oscillations, which preserve relative strength differences (Long et al. 2021; Tononi and Cirelli 2006). This process maintains overall synaptic strength at sustainable levels, preventing excessive energy consumption, cortical excitability, and reduced neural processing efficiency. In DCD, higher resting‐state delta power during wakefulness may occur because of incomplete synaptic downscaling, which may arise due to poor sleep, a problem noted in the disorder (Wiggs et al. 2016). Consequently, compensatory neural processes in the form of increased delta synchronization (i.e., increased delta power) during wakeful periods may be required to achieve the homeostatic balance typically reached during sleep. Incidentally, we can expect incomplete synaptic downscaling to result in higher cortical excitability, which should be evidenced by lower resting‐state alpha power. Thus, resting‐state delta and alpha power should be negatively correlated. An ad‐hoc analysis of our data supports this proposal, revealing a significant negative correlation between delta and alpha power (Spearman's *ρ* = −0.752, *p* < 0.001; see Figure 5). To investigate this issue further, EEG sleep studies examining delta activity in children with DCD will be required. Based on the data in this study, we predict that children with DCD would exhibit lower delta power during sleep compared to controls.

Finally, our study raises several outstanding questions that will need to be examined in future research. First, unlike Van Dyck et al. (2022), we observed significant differences between the DCD and TD groups in resting‐state power. This discrepancy may reflect differences in brain imaging modalities and analytical techniques. Van Dyck et al. (2022) employed MEG to investigate resting‐state brain activity in DCD, which offers superior spatial resolution compared to EEG. Additionally, their analyses were conducted at the source level, rather than at the electrode/sensor level. Consequently, the resting‐state power measures in the two studies are not directly comparable. The resting‐state power recorded in the current study captures more diffuse aspects of brain activity, whereas Van Dyck et al.'s (2022) measures would reflect more focal activity. Integrating the findings from both studies might suggest that resting‐state brain activity in DCD may be characterized by relatively global brain dysfunction rather than dysfunction within specific brain regions. This interpretation might explain why Van Dyck et al. (2022) observed differences between DCD and TD groups only when using functional connectivity metrics, which assess resting‐state activity on a larger spatial scale.

Second, we did not replicate findings observed in adults with DCD (Meachon et al. 2024). Evidence from other developmental disorders, however, indicates that discrepancies in resting‐state power between pediatric and adult samples are not uncommon. For example, in ADHD, lower resting‐state alpha power is reported in children, but in adults, this effect reverses or disappears (Clarke et al. 2020). Neural compensatory mechanisms (e.g., Ullman and Pullman 2015) or brain maturation (John et al. 1980) may explain these differences, necessitating longitudinal research to explore this further.

Another outstanding question concerns the specificity of resting‐state power differences to motor dysfunction. Reduced resting‐state alpha power and elevated delta power are not unique to DCD. Our findings might suggest that resting‐state delta and alpha power may serve as candidate biomarkers of motor function in DCD. However, broader correlations with attentional, cognitive, and language measures will be necessary to assess the specificity of these associations to motor impairment. For example, one or both of these frequency bands are also affected in conditions including ADHD, autism, developmental language disorder, and dyslexia (Neo et al. 2023; Newson and Thiagarajan 2019; Papagiannopoulou and Lagopoulos 2016; Stanojević et al. 2023). Thus, the differences in resting‐state power between the DCD and TD groups we observed might reflect a general marker of brain dysfunction. Another possibility is that the combination of elevated‐delta and reduced‐alpha band activity is specific to motor impairment (see Figure 4). Thus, our study may have identified resting state brain activity that is either a cause or correlate of developmental motor problems, which interestingly also occur in the aforementioned disorders (Bhat et al. 2011; Fawcett et al. 1996; Hill 2001; Kaiser et al. 2015). To better understand the role of resting‐state brain activity in neurodevelopmental disorders, future studies should correlate resting‐state data with a broader range of cognitive, language, and motor abilities to elucidate the relationship between resting‐state power and specific functional impairments.

Finally, while the results of our study, along with past research (Meachon et al. 2024; Van Dyck et al. 2022) identify atypical resting state activity in DCD, we are circumspect as to whether this is a cause or outcome of the disorder. At present, the evidence base examining resting state EEG in DCD comprises cross‐sectional designs, which, in turn, restrict the capacity to draw conclusions about the directionality or causality of the observed associations between resting‐state EEG power and motor functioning. While the findings suggest a link between neural oscillatory activity and motor ability, it remains unclear whether atypical EEG patterns contribute to poorer motor outcomes or whether motor difficulties give rise to altered patterns of neural activity. Longitudinal studies are therefore required to clarify the developmental trajectory of these associations and determine whether resting‐state EEG measures have predictive value for motor functioning over time.

## 5 Conclusion

Very little is known about resting state EEG power in children with and without DCD. The main findings to emerge from this research are that the disorder, at least in children, is characterized by reduced resting state power in the alpha band and elevated power in the delta band. At present, we suspect that abnormalities in these frequency bands indicate incomplete synaptic downscaling and elevated levels of cortical excitability. Future research is needed to examine whether this is the case and test the extent atypical alpha and delta resting state activity is specifically related to motor problems.

## Author Contributions

**Jarrad A. G. Lum:** conceptualization, data curation, formal analysis, funding acquisition, investigation, methodology, project administration, writing – original draft, writing – review and editing. **Kaila Hamilton:** methodology, project administration, writing – review and editing. **Ian Fuelscher:** conceptualization, data curation, formal analysis, funding acquisition, investigation, methodology, project administration, writing – review and editing. **Pamela Barhoun:** methodology, project administration, supervision, writing – review and editing. **Frederik J. A. Deconinck:** writing – review and editing. **Arthur De Raeve:** writing – review and editing. **Talitha C. Ford:** writing – review and editing. **Peter G. Enticott:** conceptualization, data curation, formal analysis, funding acquisition, investigation, methodology, writing – review and editing. **Jessica Waugh:** methodology, project administration. **Mugdha Mukherjee:** methodology, project administration. **Dwayne Meaney:** methodology, project administration. **Gayatri Kumar:** methodology, project administration. **Tim Silk:** conceptualization, data curation, formal analysis, funding acquisition, investigation, methodology, project administration, writing – review and editing. **Christian Hyde:** conceptualization, data curation, formal analysis, funding acquisition, investigation, methodology, project administration, writing – review and editing.

## Conflicts of Interest

The authors declare no conflicts of interest.

## Supporting information


Data S1.

## Data Availability

All data in this study are available by contacting the corresponding author upon reasonable request.
