# Supplementary material for: Resting State Electroencephalography (EEG) Reveals Atypical Oscillatory Power in Children With Development Coordination Disorder (DCD)
Source: Psychophysiology. 2025 Jun 4;62(6):e70084. doi: 10.1111/psyp.70084 (PMC12134841; doi:10.1111/psyp.70084)
Supplement: Supplementary file 1 — Data S1. [file PSYP-62-e70084-s001.docx]

**Supplementary Materials**

**Analyses of Aperiodic Activity**

Exploratory analyses examined aperiodic parameters in DCD. We tested for differences between the groups at each electrode (*n* = 17) with respect to the aperiodic offset and aperiodic slope in Eyes-Open and Eyes-Closed resting-state conditions. Differences between groups were evaluated using Wilcoxon Signed Rank Tests. The Benjamini and Hochberg (1995) False Discovery Rate (FDR) procedure was applied to the analyses for each aperiodic parameter (i.e., correcting for 17 comparisons). Summary data and FDR-corrected *p*-values for the Eyes-Open condition are presented in Table S1, and the corresponding data for the Eyes-Closed condition are in Table S2. No significant differences were observed between the groups for any of the analyses.

**Table S1.** Summary statistics and results from Wilcoxon Signed Rank Tests testing fort differences in aperiodic parameters between the TD and DCD groups with data from the Eyes-Open resting state condition.

| Electrode | Aperiodic Offset | | | | | | |  | Aperiodic Slope | | | | | | |
| --- | --- | --- | --- | --- | --- | --- | --- | --- | --- | --- | --- | --- | --- | --- | --- |
|  | TD | |  | DCD | |  | *p*-value^a^ |  | TD | |  | DCD | |  | *p*-value^a^ |
|  | *M* | *SD* |  | *M* | *SD* |  |  |  | *M* | *SD* |  | *M* | *SD* |  |  |
| F7 | 2.40 | 0.39 |  | 2.36 | 0.41 |  | .774 |  | 1.70 | 0.42 |  | 1.65 | 0.38 |  | .671 |
| F3 | 2.38 | 0.37 |  | 2.47 | 0.41 |  | .430 |  | 1.67 | 0.40 |  | 1.76 | 0.35 |  | .605 |
| Fz | 2.50 | 0.43 |  | 2.68 | 0.36 |  | .265 |  | 1.81 | 0.43 |  | 1.98 | 0.30 |  | .440 |
| F4 | 2.31 | 0.36 |  | 2.47 | 0.35 |  | .265 |  | 1.58 | 0.40 |  | 1.76 | 0.33 |  | .440 |
| F8 | 2.30 | 0.35 |  | 2.36 | 0.69 |  | .928 |  | 1.60 | 0.36 |  | 1.64 | 0.52 |  | .757 |
| T7 | 1.73 | 0.50 |  | 1.88 | 0.60 |  | .398 |  | 1.15 | 0.54 |  | 1.27 | 0.58 |  | .671 |
| C3 | 2.21 | 0.37 |  | 2.33 | 0.41 |  | .273 |  | 1.64 | 0.39 |  | 1.73 | 0.39 |  | .505 |
| Cz | 2.29 | 0.34 |  | 2.44 | 0.33 |  | .265 |  | 1.73 | 0.37 |  | 1.85 | 0.28 |  | .440 |
| C4 | 2.17 | 0.38 |  | 2.33 | 0.38 |  | .265 |  | 1.59 | 0.43 |  | 1.75 | 0.35 |  | .440 |
| T8 | 1.70 | 0.51 |  | 1.87 | 0.46 |  | .332 |  | 1.10 | 0.54 |  | 1.21 | 0.47 |  | .671 |
| P7 | 1.81 | 0.51 |  | 1.92 | 0.44 |  | .436 |  | 1.40 | 0.51 |  | 1.47 | 0.38 |  | .757 |
| P3 | 2.13 | 0.35 |  | 2.26 | 0.38 |  | .273 |  | 1.62 | 0.37 |  | 1.71 | 0.33 |  | .627 |
| Pz | 2.21 | 0.36 |  | 2.35 | 0.36 |  | .265 |  | 1.70 | 0.39 |  | 1.79 | 0.31 |  | .605 |
| P4 | 2.14 | 0.39 |  | 2.30 | 0.38 |  | .265 |  | 1.62 | 0.41 |  | 1.75 | 0.34 |  | .440 |
| P8 | 1.89 | 0.38 |  | 2.06 | 0.43 |  | .265 |  | 1.42 | 0.44 |  | 1.59 | 0.39 |  | .440 |
| O1 | 2.01 | 0.48 |  | 2.19 | 0.45 |  | .265 |  | 1.47 | 0.48 |  | 1.57 | 0.42 |  | .671 |
| O2 | 2.05 | 0.41 |  | 2.13 | 0.46 |  | .430 |  | 1.49 | 0.41 |  | 1.50 | 0.47 |  | .757 |

^a^*p*-values corrected for multiple comparisons using Benjamini and Hochberg (1995) False Discovery Rate procedure.

**Table S2.** Summary statistics and results from Wilcoxon Signed Rank Tests testing fort differences in aperiodic parameters between the TD and DCD groups with data from the Eyes-Closed resting state condition.

| Electrode | Aperiodic Offset | | | | | | |  | Aperiodic Slope | | | | | | |
| --- | --- | --- | --- | --- | --- | --- | --- | --- | --- | --- | --- | --- | --- | --- | --- |
|  | TD | |  | DCD | |  | *p*-value^a^ |  | TD | |  | DCD | |  | *p*-value^a^ |
|  | *M* | *SD* |  | *M* | *SD* |  |  |  | *M* | *SD* |  | *M* | *SD* |  |  |
| F7 | 2.58 | 0.44 |  | 2.55 | 0.44 |  | .980 |  | 1.87 | 0.36 |  | 1.84 | 0.36 |  | .957 |
| F3 | 2.50 | 0.35 |  | 2.48 | 0.35 |  | .980 |  | 1.82 | 0.30 |  | 1.80 | 0.30 |  | .957 |
| Fz | 2.61 | 0.43 |  | 2.59 | 0.43 |  | .980 |  | 1.90 | 0.34 |  | 1.89 | 0.34 |  | .957 |
| F4 | 2.47 | 0.35 |  | 2.46 | 0.35 |  | .980 |  | 1.79 | 0.27 |  | 1.77 | 0.27 |  | .957 |
| F8 | 2.46 | 0.50 |  | 2.54 | 0.50 |  | .980 |  | 1.79 | 0.43 |  | 1.85 | 0.43 |  | .957 |
| T7 | 1.97 | 0.43 |  | 1.95 | 0.43 |  | .980 |  | 1.40 | 0.37 |  | 1.40 | 0.37 |  | .957 |
| C3 | 2.34 | 0.39 |  | 2.32 | 0.39 |  | .980 |  | 1.73 | 0.32 |  | 1.71 | 0.32 |  | .957 |
| Cz | 2.43 | 0.37 |  | 2.41 | 0.37 |  | .980 |  | 1.82 | 0.30 |  | 1.79 | 0.30 |  | .957 |
| C4 | 2.31 | 0.39 |  | 2.32 | 0.39 |  | .980 |  | 1.71 | 0.31 |  | 1.70 | 0.31 |  | .957 |
| T8 | 1.95 | 0.52 |  | 1.95 | 0.52 |  | .980 |  | 1.36 | 0.45 |  | 1.36 | 0.45 |  | .957 |
| P7 | 2.02 | 0.43 |  | 2.04 | 0.43 |  | .980 |  | 1.58 | 0.32 |  | 1.54 | 0.32 |  | .957 |
| P3 | 2.29 | 0.40 |  | 2.30 | 0.40 |  | .980 |  | 1.73 | 0.31 |  | 1.70 | 0.31 |  | .957 |
| Pz | 2.38 | 0.41 |  | 2.37 | 0.41 |  | .980 |  | 1.81 | 0.34 |  | 1.76 | 0.34 |  | .957 |
| P4 | 2.34 | 0.38 |  | 2.28 | 0.38 |  | .980 |  | 1.76 | 0.28 |  | 1.67 | 0.28 |  | .957 |
| P8 | 2.16 | 0.49 |  | 2.13 | 0.49 |  | .980 |  | 1.63 | 0.33 |  | 1.53 | 0.33 |  | .957 |
| O1 | 2.23 | 0.54 |  | 2.18 | 0.54 |  | .980 |  | 1.58 | 0.42 |  | 1.52 | 0.42 |  | .957 |
| O2 | 2.23 | 0.48 |  | 2.11 | 0.48 |  | .980 |  | 1.58 | 0.36 |  | 1.43 | 0.36 |  | .957 |

^a^*p*-values corrected for multiple comparisons using Benjamini and Hochberg (1995) False Discovery Rate procedure.

**Table S3.** Summary data showing means, standard deviations and range for resting state power (in μV^2^) in the eyes open condition in all studied frequency bands reported by group.

|  | Delta | | | | | | | | |
| --- | --- | --- | --- | --- | --- | --- | --- | --- | --- |
|  | TD | | | |  | DCD | | | |
| Electrode | M | SD | Min | Max |  | M | SD | Min | Max |
| F7 | 0.326 | 0.261 | -0.048 | 1.180 |  | 0.392 | 0.225 | -0.011 | 0.878 |
| F3 | 0.188 | 0.249 | -0.075 | 1.066 |  | 0.246 | 0.228 | -0.031 | 0.860 |
| Fz | 0.084 | 0.169 | -0.182 | 0.803 |  | 0.240 | 0.250 | -0.023 | 0.946 |
| F4 | 0.185 | 0.216 | -0.077 | 0.947 |  | 0.242 | 0.203 | -0.028 | 0.736 |
| F8 | 0.308 | 0.238 | -0.059 | 0.900 |  | 0.438 | 0.253 | -0.004 | 1.075 |
| T7 | 0.463 | 0.357 | -0.009 | 1.245 |  | 0.463 | 0.232 | 0.095 | 1.064 |
| C3 | 0.107 | 0.205 | -0.150 | 0.843 |  | 0.172 | 0.206 | -0.024 | 0.670 |
| Cz | 0.053 | 0.116 | -0.119 | 0.505 |  | 0.143 | 0.200 | -0.051 | 0.643 |
| C4 | 0.063 | 0.151 | -0.139 | 0.843 |  | 0.151 | 0.156 | -0.087 | 0.520 |
| T8 | 0.440 | 0.316 | -0.007 | 1.286 |  | 0.584 | 0.242 | 0.198 | 1.118 |
| P7 | 0.164 | 0.206 | -0.097 | 0.823 |  | 0.252 | 0.235 | -0.076 | 0.869 |
| P3 | 0.058 | 0.125 | -0.125 | 0.425 |  | 0.126 | 0.176 | -0.061 | 0.499 |
| Pz | 0.042 | 0.141 | -0.117 | 0.694 |  | 0.088 | 0.135 | -0.053 | 0.492 |
| P4 | 0.051 | 0.133 | -0.114 | 0.592 |  | 0.124 | 0.163 | -0.065 | 0.480 |
| P8 | 0.149 | 0.190 | -0.052 | 0.753 |  | 0.220 | 0.203 | -0.055 | 0.626 |
| O1 | 0.102 | 0.173 | -0.066 | 0.750 |  | 0.206 | 0.205 | -0.058 | 0.718 |
| O2 | 0.098 | 0.135 | -0.080 | 0.546 |  | 0.213 | 0.215 | -0.026 | 0.866 |
|  |  |  |  |  |  |  |  |  |  |
|  | Theta | | | | | | | | |
|  | TD | | | |  | DCD | | | |
| Electrode | M | SD | Min | Max |  | M | SD | Min | Max |
| F7 | 0.077 | 0.169 | -0.151 | 0.618 |  | 0.081 | 0.255 | -0.236 | 0.972 |
| F3 | 0.151 | 0.153 | -0.196 | 0.566 |  | 0.178 | 0.268 | -0.162 | 1.103 |
| Fz | 0.182 | 0.143 | -0.121 | 0.599 |  | 0.224 | 0.278 | -0.152 | 1.038 |
| F4 | 0.151 | 0.157 | -0.157 | 0.544 |  | 0.161 | 0.272 | -0.167 | 1.140 |
| F8 | 0.073 | 0.153 | -0.223 | 0.490 |  | 0.100 | 0.234 | -0.294 | 0.717 |
| T7 | 0.107 | 0.200 | -0.208 | 0.864 |  | 0.079 | 0.300 | -0.199 | 1.049 |
| C3 | 0.207 | 0.161 | -0.305 | 0.609 |  | 0.214 | 0.276 | -0.110 | 1.165 |
| Cz | 0.262 | 0.139 | -0.016 | 0.572 |  | 0.280 | 0.289 | -0.120 | 1.150 |
| C4 | 0.180 | 0.150 | -0.114 | 0.573 |  | 0.175 | 0.263 | -0.149 | 1.005 |
| T8 | 0.047 | 0.169 | -0.289 | 0.400 |  | 0.073 | 0.273 | -0.359 | 0.984 |
| P7 | 0.095 | 0.163 | -0.382 | 0.545 |  | 0.122 | 0.256 | -0.204 | 1.016 |
| P3 | 0.181 | 0.167 | -0.186 | 0.586 |  | 0.183 | 0.271 | -0.144 | 0.984 |
| Pz | 0.229 | 0.178 | -0.108 | 0.674 |  | 0.223 | 0.296 | -0.127 | 1.071 |
| P4 | 0.169 | 0.165 | -0.186 | 0.652 |  | 0.167 | 0.277 | -0.172 | 0.985 |
| P8 | 0.109 | 0.159 | -0.176 | 0.613 |  | 0.125 | 0.257 | -0.209 | 0.920 |
| O1 | 0.131 | 0.168 | -0.216 | 0.626 |  | 0.140 | 0.274 | -0.136 | 0.988 |
| O2 | 0.113 | 0.182 | -0.231 | 0.675 |  | 0.126 | 0.263 | -0.302 | 0.812 |
|  |  |  |  |  |  |  |  |  |  |
|  | Alpha | | | | | | | | |
|  | TD | | | |  | DCD | | | |
| Electrode | M | SD | Min | Max |  | M | SD | Min | Max |
| F7 | -0.028 | 0.195 | -0.409 | 0.599 |  | -0.099 | 0.140 | -0.340 | 0.188 |
| F3 | 0.099 | 0.157 | -0.225 | 0.564 |  | 0.032 | 0.127 | -0.177 | 0.282 |
| Fz | 0.150 | 0.188 | -0.105 | 0.709 |  | 0.083 | 0.148 | -0.200 | 0.355 |
| F4 | 0.087 | 0.176 | -0.307 | 0.690 |  | 0.027 | 0.132 | -0.319 | 0.275 |
| F8 | -0.009 | 0.189 | -0.424 | 0.544 |  | -0.058 | 0.125 | -0.342 | 0.134 |
| T7 | 0.022 | 0.178 | -0.317 | 0.559 |  | -0.107 | 0.161 | -0.476 | 0.231 |
| C3 | 0.314 | 0.221 | -0.055 | 0.889 |  | 0.164 | 0.155 | -0.082 | 0.485 |
| Cz | 0.334 | 0.217 | -0.024 | 1.027 |  | 0.223 | 0.167 | -0.062 | 0.508 |
| C4 | 0.297 | 0.234 | -0.192 | 0.961 |  | 0.162 | 0.168 | -0.148 | 0.526 |
| T8 | -0.013 | 0.190 | -0.362 | 0.690 |  | -0.123 | 0.182 | -0.570 | 0.210 |
| P7 | 0.228 | 0.235 | -0.206 | 0.882 |  | 0.107 | 0.179 | -0.193 | 0.526 |
| P3 | 0.397 | 0.258 | -0.068 | 1.035 |  | 0.241 | 0.190 | -0.053 | 0.675 |
| Pz | 0.468 | 0.259 | -0.020 | 1.136 |  | 0.276 | 0.203 | -0.069 | 0.693 |
| P4 | 0.440 | 0.263 | -0.105 | 1.151 |  | 0.246 | 0.195 | -0.088 | 0.669 |
| P8 | 0.286 | 0.230 | -0.090 | 0.845 |  | 0.129 | 0.197 | -0.162 | 0.645 |
| O1 | 0.441 | 0.294 | -0.016 | 1.245 |  | 0.255 | 0.201 | -0.106 | 0.665 |
| O2 | 0.426 | 0.275 | 0.002 | 1.147 |  | 0.224 | 0.195 | -0.095 | 0.587 |
|  |  |  |  |  |  |  |  |  |  |
|  |  |  |  |  |  |  |  |  |  |
|  | Gamma 1 | | | | | | | | |
|  | TD | | | |  | DCD | | | |
| Electrode | M | SD | Min | Max |  | M | SD | Min | Max |
| F7 | 0.168 | 0.127 | -0.037 | 0.453 |  | 0.258 | 0.141 | 0.032 | 0.497 |
| F3 | 0.095 | 0.106 | -0.045 | 0.401 |  | 0.130 | 0.111 | -0.014 | 0.442 |
| Fz | 0.070 | 0.093 | -0.066 | 0.334 |  | 0.128 | 0.129 | -0.025 | 0.556 |
| F4 | 0.090 | 0.106 | -0.037 | 0.438 |  | 0.161 | 0.137 | 0.007 | 0.554 |
| F8 | 0.194 | 0.163 | -0.019 | 0.651 |  | 0.224 | 0.136 | 0.035 | 0.562 |
| T7 | 0.188 | 0.144 | -0.010 | 0.498 |  | 0.217 | 0.113 | 0.053 | 0.471 |
| C3 | 0.088 | 0.109 | -0.042 | 0.339 |  | 0.137 | 0.124 | -0.008 | 0.538 |
| Cz | 0.050 | 0.095 | -0.090 | 0.309 |  | 0.101 | 0.122 | -0.023 | 0.547 |
| C4 | 0.095 | 0.128 | -0.043 | 0.568 |  | 0.147 | 0.121 | -0.026 | 0.491 |
| T8 | 0.188 | 0.136 | -0.011 | 0.647 |  | 0.210 | 0.126 | 0.042 | 0.565 |
| P7 | 0.147 | 0.134 | -0.033 | 0.531 |  | 0.166 | 0.123 | -0.034 | 0.471 |
| P3 | 0.085 | 0.112 | -0.036 | 0.338 |  | 0.125 | 0.117 | -0.036 | 0.490 |
| Pz | 0.068 | 0.106 | -0.043 | 0.342 |  | 0.115 | 0.124 | -0.035 | 0.522 |
| P4 | 0.073 | 0.114 | -0.043 | 0.477 |  | 0.128 | 0.113 | -0.035 | 0.483 |
| P8 | 0.132 | 0.128 | -0.036 | 0.479 |  | 0.184 | 0.123 | -0.019 | 0.483 |
| O1 | 0.098 | 0.112 | -0.040 | 0.426 |  | 0.148 | 0.140 | -0.033 | 0.563 |
| O2 | 0.113 | 0.113 | -0.054 | 0.408 |  | 0.176 | 0.124 | -0.018 | 0.474 |
|  |  |  |  |  |  |  |  |  |  |
|  | Gamma 2 | | | | | | | | |
|  | TD | | | |  | DCD | | | |
| Electrode | M | SD | Min | Max |  | M | SD | Min | Max |
| F7 | 0.168 | 0.127 | -0.037 | 0.453 |  | 0.258 | 0.141 | 0.032 | 0.497 |
| F3 | 0.095 | 0.106 | -0.045 | 0.401 |  | 0.130 | 0.111 | -0.014 | 0.442 |
| Fz | 0.070 | 0.093 | -0.066 | 0.334 |  | 0.128 | 0.129 | -0.025 | 0.556 |
| F4 | 0.090 | 0.106 | -0.037 | 0.438 |  | 0.161 | 0.137 | 0.007 | 0.554 |
| F8 | 0.194 | 0.163 | -0.019 | 0.651 |  | 0.224 | 0.136 | 0.035 | 0.562 |
| T7 | 0.188 | 0.144 | -0.010 | 0.498 |  | 0.217 | 0.113 | 0.053 | 0.471 |
| C3 | 0.088 | 0.109 | -0.042 | 0.339 |  | 0.137 | 0.124 | -0.008 | 0.538 |
| Cz | 0.050 | 0.095 | -0.090 | 0.309 |  | 0.101 | 0.122 | -0.023 | 0.547 |
| C4 | 0.095 | 0.128 | -0.043 | 0.568 |  | 0.147 | 0.121 | -0.026 | 0.491 |
| T8 | 0.188 | 0.136 | -0.011 | 0.647 |  | 0.210 | 0.126 | 0.042 | 0.565 |
| P7 | 0.147 | 0.134 | -0.033 | 0.531 |  | 0.166 | 0.123 | -0.034 | 0.471 |
| P3 | 0.085 | 0.112 | -0.036 | 0.338 |  | 0.125 | 0.117 | -0.036 | 0.490 |
| Pz | 0.068 | 0.106 | -0.043 | 0.342 |  | 0.115 | 0.124 | -0.035 | 0.522 |
| P4 | 0.073 | 0.114 | -0.043 | 0.477 |  | 0.128 | 0.113 | -0.035 | 0.483 |
| P8 | 0.132 | 0.128 | -0.036 | 0.479 |  | 0.184 | 0.123 | -0.019 | 0.483 |
| O1 | 0.098 | 0.112 | -0.040 | 0.426 |  | 0.148 | 0.140 | -0.033 | 0.563 |
| O2 | 0.113 | 0.113 | -0.054 | 0.408 |  | 0.176 | 0.124 | -0.018 | 0.474 |

**Table S4**. FDR corrected *p*-values testing for differences between TD and DCD groups at all frequency bands and electrodes in the Eyes-Open condition. Significant differences (*p* < .05) are highlighted in bold.

| Electrode | FDR Corrected *p*-values eyes open condition | | | | |
| --- | --- | --- | --- | --- | --- |
|  | Delta | Theta | Alpha | Beta | Gamma |
| F7 | .081 | .922 | .132 | .916 | .061 |
| F3 | .162 | .974 | .132 | .611 | .199 |
| Fz | .034 | .974 | .342 | .423 | .061 |
| F4 | .162 | .922 | .124 | .611 | .061 |
| F8 | **.040** | .974 | .350 | .611 | .340 |
| T7 | .498 | .761 | **.010** | .611 | .155 |
| C3 | .068 | .761 | **.008** | .611 | .061 |
| Cz | .069 | .922 | .078 | .611 | .087 |
| C4 | **.034** | .761 | **.013** | .611 | .062 |
| T8 | .051 | .974 | **.025** | .709 | .367 |
| P7 | .078 | .974 | **.031** | .614 | .596 |
| P3 | .106 | .761 | **.017** | .611 | .199 |
| Pz | .067 | .761 | **.008** | .423 | .215 |
| P4 | .054 | .761 | **.008** | .423 | .061 |
| P8 | .081 | .974 | **.008** | .725 | .087 |
| O1 | **.038** | .761 | **.008** | .611 | .199 |
| O2 | **.038** | .922 | **.008** | .725 | .061 |

**Table S5.** Summary data showing means, standard deviations and range for resting state power (in μV^2^) in the eyes closed condition in all studied frequency bands reported by group.

|  | Delta | | | | | | | | |
| --- | --- | --- | --- | --- | --- | --- | --- | --- | --- |
|  | TD | | | |  | DCD | | | |
| Electrode | M | SD | Min | Max |  | M | SD | Min | Max |
| F7 | 0.262 | 0.266 | -0.045 | 1.566 |  | 0.340 | 0.215 | -0.028 | 0.896 |
| F3 | 0.066 | 0.147 | -0.123 | 0.641 |  | 0.180 | 0.201 | -0.073 | 0.736 |
| Fz | 0.067 | 0.186 | -0.123 | 0.787 |  | 0.134 | 0.146 | -0.101 | 0.415 |
| F4 | 0.069 | 0.171 | -0.123 | 0.755 |  | 0.200 | 0.215 | -0.077 | 0.756 |
| F8 | 0.252 | 0.191 | -0.076 | 0.797 |  | 0.409 | 0.257 | 0.043 | 1.097 |
| T7 | 0.316 | 0.334 | -0.070 | 1.682 |  | 0.449 | 0.350 | 0.020 | 1.320 |
| C3 | 0.031 | 0.115 | -0.124 | 0.540 |  | 0.122 | 0.164 | -0.073 | 0.528 |
| Cz | 0.005 | 0.083 | -0.161 | 0.379 |  | 0.063 | 0.123 | -0.074 | 0.373 |
| C4 | 0.044 | 0.135 | -0.116 | 0.565 |  | 0.111 | 0.171 | -0.134 | 0.572 |
| T8 | 0.284 | 0.263 | -0.042 | 0.870 |  | 0.492 | 0.285 | 0.032 | 1.184 |
| P7 | 0.067 | 0.122 | -0.086 | 0.417 |  | 0.156 | 0.183 | -0.072 | 0.514 |
| P3 | 0.007 | 0.090 | -0.135 | 0.395 |  | 0.077 | 0.141 | -0.140 | 0.403 |
| Pz | 0.008 | 0.125 | -0.135 | 0.726 |  | 0.059 | 0.130 | -0.096 | 0.411 |
| P4 | 0.000 | 0.125 | -0.134 | 0.755 |  | 0.066 | 0.122 | -0.145 | 0.336 |
| P8 | 0.057 | 0.113 | -0.114 | 0.450 |  | 0.175 | 0.185 | -0.127 | 0.657 |
| O1 | 0.057 | 0.127 | -0.129 | 0.543 |  | 0.145 | 0.178 | -0.115 | 0.508 |
| O2 | 0.052 | 0.125 | -0.199 | 0.443 |  | 0.150 | 0.149 | -0.066 | 0.489 |
|  |  |  |  |  |  |  |  |  |  |
|  | Theta | | | | | | | | |
|  | TD | | | |  | DCD | | | |
| Electrode | M | SD | Min | Max |  | M | SD | Min | Max |
| F7 | 0.058 | 0.186 | -0.211 | 0.884 |  | 0.008 | 0.218 | -0.271 | 0.527 |
| F3 | 0.178 | 0.156 | -0.160 | 0.618 |  | 0.175 | 0.275 | -0.251 | 1.045 |
| Fz | 0.224 | 0.169 | -0.102 | 0.700 |  | 0.196 | 0.280 | -0.241 | 1.001 |
| F4 | 0.187 | 0.165 | -0.141 | 0.734 |  | 0.165 | 0.279 | -0.283 | 1.065 |
| F8 | 0.038 | 0.160 | -0.172 | 0.534 |  | 0.039 | 0.231 | -0.365 | 0.600 |
| T7 | 0.120 | 0.230 | -0.240 | 1.103 |  | 0.125 | 0.348 | -0.263 | 1.230 |
| C3 | 0.240 | 0.170 | -0.148 | 0.747 |  | 0.210 | 0.292 | -0.212 | 1.060 |
| Cz | 0.325 | 0.173 | -0.077 | 0.749 |  | 0.273 | 0.280 | -0.158 | 1.053 |
| C4 | 0.249 | 0.167 | -0.103 | 0.768 |  | 0.194 | 0.275 | -0.227 | 0.944 |
| T8 | 0.083 | 0.169 | -0.181 | 0.464 |  | 0.103 | 0.317 | -0.417 | 0.981 |
| P7 | 0.192 | 0.196 | -0.169 | 0.848 |  | 0.166 | 0.306 | -0.191 | 1.186 |
| P3 | 0.261 | 0.193 | -0.101 | 0.748 |  | 0.214 | 0.290 | -0.149 | 1.037 |
| Pz | 0.329 | 0.205 | -0.042 | 0.861 |  | 0.248 | 0.303 | -0.147 | 1.067 |
| P4 | 0.267 | 0.193 | -0.051 | 0.866 |  | 0.207 | 0.273 | -0.137 | 0.962 |
| P8 | 0.220 | 0.198 | -0.116 | 0.820 |  | 0.179 | 0.288 | -0.188 | 1.014 |
| O1 | 0.201 | 0.205 | -0.131 | 0.771 |  | 0.179 | 0.347 | -0.282 | 1.089 |
| O2 | 0.190 | 0.215 | -0.165 | 0.775 |  | 0.141 | 0.304 | -0.310 | 0.954 |
|  |  |  |  |  |  |  |  |  |  |
|  | Alpha | | | | | | | | |
|  | TD | | | |  | DCD | | | |
| Electrode | M | SD | Min | Max |  | M | SD | Min | Max |
| F7 | 0.192 | 0.193 | -0.232 | 0.663 |  | 0.040 | 0.240 | -0.369 | 0.446 |
| F3 | 0.347 | 0.215 | 0.006 | 0.925 |  | 0.226 | 0.187 | -0.064 | 0.614 |
| Fz | 0.366 | 0.216 | -0.180 | 0.971 |  | 0.243 | 0.195 | -0.067 | 0.609 |
| F4 | 0.340 | 0.203 | -0.013 | 0.968 |  | 0.219 | 0.172 | -0.100 | 0.595 |
| F8 | 0.166 | 0.193 | -0.354 | 0.691 |  | 0.069 | 0.182 | -0.282 | 0.382 |
| T7 | 0.230 | 0.216 | -0.185 | 0.771 |  | 0.087 | 0.200 | -0.424 | 0.481 |
| C3 | 0.500 | 0.244 | 0.090 | 1.012 |  | 0.339 | 0.197 | 0.027 | 0.782 |
| Cz | 0.564 | 0.236 | 0.108 | 1.123 |  | 0.427 | 0.207 | 0.073 | 0.919 |
| C4 | 0.499 | 0.240 | 0.078 | 1.074 |  | 0.341 | 0.208 | 0.037 | 0.794 |
| T8 | 0.163 | 0.227 | -0.351 | 0.601 |  | 0.054 | 0.204 | -0.450 | 0.390 |
| P7 | 0.535 | 0.279 | -0.167 | 1.225 |  | 0.370 | 0.274 | -0.008 | 0.933 |
| P3 | 0.660 | 0.273 | 0.130 | 1.361 |  | 0.525 | 0.266 | 0.034 | 1.031 |
| Pz | 0.746 | 0.277 | 0.041 | 1.346 |  | 0.567 | 0.258 | 0.072 | 1.040 |
| P4 | 0.755 | 0.291 | 0.125 | 1.330 |  | 0.560 | 0.304 | 0.038 | 1.103 |
| P8 | 0.635 | 0.306 | 0.016 | 1.191 |  | 0.450 | 0.313 | -0.022 | 1.077 |
| O1 | 0.740 | 0.317 | 0.089 | 1.508 |  | 0.564 | 0.291 | 0.022 | 1.035 |
| O2 | 0.731 | 0.317 | 0.044 | 1.564 |  | 0.520 | 0.304 | -0.066 | 1.110 |
|  |  |  |  |  |  |  |  |  |  |
|  |  |  |  |  |  |  |  |  |  |
|  | Gamma 1 | | | | | | | | |
|  | TD | | | |  | DCD | | | |
| Electrode | M | SD | Min | Max |  | M | SD | Min | Max |
| F7 | 0.155 | 0.136 | -0.021 | 0.582 |  | 0.254 | 0.170 | 0.010 | 0.646 |
| F3 | 0.089 | 0.103 | -0.049 | 0.348 |  | 0.139 | 0.126 | -0.016 | 0.482 |
| Fz | 0.068 | 0.093 | -0.069 | 0.288 |  | 0.115 | 0.111 | -0.031 | 0.321 |
| F4 | 0.066 | 0.087 | -0.045 | 0.290 |  | 0.139 | 0.121 | -0.023 | 0.466 |
| F8 | 0.198 | 0.150 | -0.025 | 0.662 |  | 0.258 | 0.148 | 0.026 | 0.624 |
| T7 | 0.205 | 0.140 | -0.028 | 0.542 |  | 0.215 | 0.145 | 0.005 | 0.569 |
| C3 | 0.082 | 0.119 | -0.063 | 0.507 |  | 0.125 | 0.135 | -0.043 | 0.513 |
| Cz | 0.053 | 0.100 | -0.056 | 0.349 |  | 0.094 | 0.113 | -0.043 | 0.326 |
| C4 | 0.086 | 0.105 | -0.042 | 0.393 |  | 0.140 | 0.120 | -0.034 | 0.427 |
| T8 | 0.209 | 0.138 | -0.003 | 0.558 |  | 0.222 | 0.142 | 0.001 | 0.588 |
| P7 | 0.122 | 0.140 | -0.049 | 0.481 |  | 0.141 | 0.111 | -0.013 | 0.374 |
| P3 | 0.060 | 0.103 | -0.049 | 0.386 |  | 0.083 | 0.101 | -0.038 | 0.275 |
| Pz | 0.043 | 0.085 | -0.055 | 0.349 |  | 0.080 | 0.088 | -0.037 | 0.242 |
| P4 | 0.050 | 0.090 | -0.069 | 0.323 |  | 0.086 | 0.101 | -0.041 | 0.269 |
| P8 | 0.107 | 0.135 | -0.039 | 0.511 |  | 0.140 | 0.124 | -0.036 | 0.387 |
| O1 | 0.075 | 0.110 | -0.054 | 0.401 |  | 0.092 | 0.110 | -0.040 | 0.311 |
| O2 | 0.087 | 0.119 | -0.068 | 0.414 |  | 0.091 | 0.102 | -0.025 | 0.356 |
|  |  |  |  |  |  |  |  |  |  |
|  | Gamma 2 | | | | | | | | |
|  | TD | | | |  | DCD | | | |
| Electrode | M | SD | Min | Max |  | M | SD | Min | Max |
| F7 | 0.051 | 0.048 | -0.034 | 0.286 |  | 0.049 | 0.033 | -0.021 | 0.131 |
| F3 | 0.039 | 0.030 | -0.007 | 0.145 |  | 0.043 | 0.031 | -0.013 | 0.105 |
| Fz | 0.032 | 0.030 | -0.019 | 0.132 |  | 0.026 | 0.038 | -0.030 | 0.126 |
| F4 | 0.050 | 0.025 | 0.006 | 0.101 |  | 0.054 | 0.050 | -0.015 | 0.183 |
| F8 | 0.060 | 0.044 | -0.007 | 0.204 |  | 0.054 | 0.043 | -0.038 | 0.169 |
| T7 | 0.051 | 0.041 | -0.019 | 0.227 |  | 0.044 | 0.018 | 0.013 | 0.101 |
| C3 | 0.052 | 0.041 | 0.001 | 0.223 |  | 0.047 | 0.030 | 0.005 | 0.117 |
| Cz | 0.039 | 0.026 | -0.010 | 0.131 |  | 0.041 | 0.031 | -0.016 | 0.151 |
| C4 | 0.049 | 0.032 | -0.012 | 0.150 |  | 0.046 | 0.038 | -0.019 | 0.197 |
| T8 | 0.065 | 0.043 | 0.002 | 0.250 |  | 0.049 | 0.048 | -0.046 | 0.237 |
| P7 | 0.048 | 0.033 | 0.009 | 0.139 |  | 0.056 | 0.036 | -0.011 | 0.147 |
| P3 | 0.041 | 0.025 | -0.004 | 0.147 |  | 0.039 | 0.025 | -0.014 | 0.100 |
| Pz | 0.038 | 0.022 | -0.027 | 0.103 |  | 0.041 | 0.030 | -0.002 | 0.150 |
| P4 | 0.039 | 0.019 | 0.000 | 0.092 |  | 0.041 | 0.036 | -0.018 | 0.179 |
| P8 | 0.037 | 0.026 | -0.008 | 0.152 |  | 0.040 | 0.033 | -0.016 | 0.180 |
| O1 | 0.037 | 0.017 | -0.004 | 0.092 |  | 0.040 | 0.027 | -0.005 | 0.123 |
| O2 | 0.037 | 0.019 | 0.007 | 0.129 |  | 0.036 | 0.023 | 0.001 | 0.101 |

**Table S6.** FDR corrected *p*-values testing for differences between TD and DCD groups at all frequency bands and electrodes in the Eyes-Closed condition. Significant differences (*p* < .05) are highlighted in bold.

| Electrode | FDR Corrected *p*-values eyes closed condition | | | | |
| --- | --- | --- | --- | --- | --- |
|  | Delta | Theta | Alpha | Beta | Gamma |
| F7 | **.041** | .192 | **.020** | .584 | .079 |
| F3 | **.009** | .400 | **.020** | .469 | .144 |
| Fz | **.009** | .252 | **.023** | .469 | .144 |
| F4 | **.006** | .252 | **.020** | .469 | .079 |
| F8 | **.009** | .530 | **.042** | .826 | .251 |
| T7 | .053 | .535 | **.020** | .584 | .939 |
| C3 | **.016** | .233 | **.020** | .615 | .291 |
| Cz | **.043** | .192 | **.020** | .469 | .144 |
| C4 | **.041** | .182 | **.020** | .584 | .144 |
| T8 | **.009** | .796 | **.040** | .570 | .629 |
| P7 | **.030** | .252 | **.020** | .584 | .244 |
| P3 | **.026** | .192 | **.042** | .469 | .291 |
| Pz | **.043** | .182 | **.020** | .469 | .144 |
| P4 | **.009** | .182 | **.020** | .469 | .144 |
| P8 | **.010** | .252 | **.020** | .469 | .164 |
| O1 | **.043** | .400 | **.022** | .469 | .256 |
| O2 | **.009** | .252 | **.020** | .469 | .337 |
